# Compressibility of Lithium Hexafluorophosphate Solutions in Two Carbonate Solvents

**DOI:** 10.1021/acs.jced.2c00711

**Published:** 2023-03-14

**Authors:** Andrew A. Wang, Delia Persa, Sara Helin, Kirk P. Smith, Jason L. Raymond, Charles W. Monroe

**Affiliations:** † Department of Engineering Science, University of Oxford, Parks Road, Oxford OX1 3PJ, U.K.; ‡ The Faraday Institution, Becquerel Avenue, Harwell Campus, Didcot OX11 0RA, U.K.

## Abstract

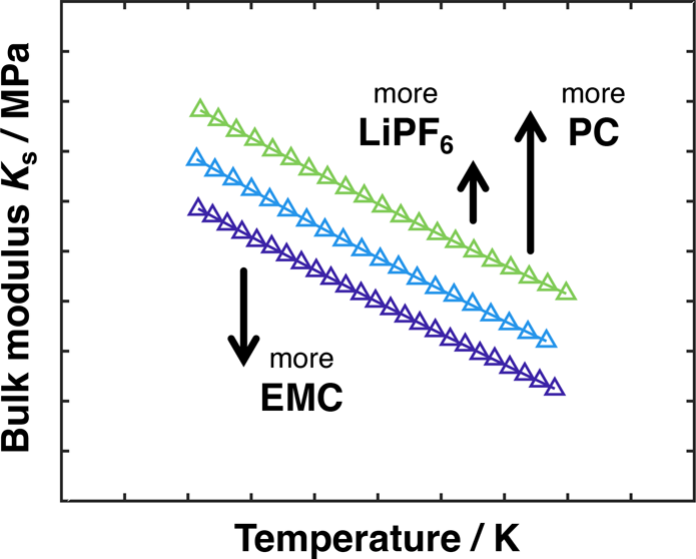

Speed-of-sound measurements are performed to establish how the isentropic bulk modulus *K*_s_ of the electrolyte system comprising lithium hexafluorophospate (LiPF_6_) in blends of propylene carbonate (PC) and ethyl methyl carbonate (EMC) varies with salt molality *m*, mass fraction of PC in the PC:EMC cosolvent *f*, and temperature *T*. Bulk moduli are calculated by combining acoustic time-of-flight data between parallel walls of a liquid-filled cuvette with densitometric data for a sequence of binary and ternary salt solutions. Correlations are presented to yield *K*_s_ (*m*, *f*, *T*) accurately for nine compositions spanning the range *m* = 0–2 mol kg^–1^ and *f* = 0–1, at temperatures *T* ranging from 283.15 to 313.15 K. Electrolyte compressibility varies most with solvent ratio, followed by salt content and temperature, with *K*_s_ ranging from 1 to 3 GPa. Composition-dependent acoustical properties elucidate the nature of speciation and solvation states in bulk electrolytes, and could be useful to identify the features of individual phases within solution-permeated porous electrodes.

## Introduction

The monitoring of electrochemical devices during their operation and manufacture increasingly relies on acoustical techniques.^1^ Touted for being noninvasive, inexpensive, and rapid, acoustical approaches have garnered substantial scientific and commercial consideration.^2^ Ultrasonic acoustic waves have been applied to lithium-ion batteries to evaluate the state of charge and probe degradation phenomena.^3−8^ They have also been used to track internal defects,^9^ the drying of electrode slurry coatings,^10^ electrolyte infiltration and wetting,^11^ and the postformation break-in period of new batteries.^12^

The studies mentioned above exploit the differences in mechanical properties of battery materials as they transmit and reflect acoustic signals. For example, the elastic modulus *E* and density ρ can differ significantly between solid active materials such as lithium cobalt oxide (*E* ≈ 190 GPa, ρ ≈ 4.8 g.cm^–3^)^13^ and pure graphite (*E* ≈ 30 GPa, ρ ≈ 2.3 g.cm^–3^),^14^ leading to differences in the speeds at which longitudinal sound waves move through them. The properties of active-material particles also vary with state of charge, with the modulus of graphite changing by a factor of three as lithiation progresses.^15,16^

A typical electrode composite in a lithium-ion cell consists of a packed solid layer containing active particles, conductive additives, and adhesive binder, whose pore structure is permeated by a liquid electrolyte. Acoustic measurements by Chang and colleagues of electrolyte-wetted electrode composites showed an effective stiffness much lower than expected from a volume-weighted average of the liquid/solid matrix.^17^ Biot’s theory of wave propagation through fluid-saturated porous media can be used to resolve signal contributions from interpenetrating fluid and solid phases.^18,19^ This approach requires knowledge of the electrolyte solution’s bulk modulus, however, which researchers have typically estimated using property values for neat solvents.^5,20,21^

The speed of sound *c* and isentropic bulk modulus (or inverse isentropic compressibility) *K*_s_ are commonly reported thermodynamic properties of solutions.^22−24^ Given the solution’s density ρ, the Newton–Laplace equation
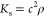
1relates the two properties. Despite their ubiquity in continuum mechanics, *c* and *K*_s_ values relevant to nonaqueous electrolytes for lithium-ion batteries are scarcely reported.^25−28^ Sound speed or compressibility measurements for battery electrolytes containing lithium hexafluorophosphate (LiPF_6_) are notably lacking.

Lithium battery electrolytes based on LiPF_6_ are typically formulated with mixtures of linear and cyclic carbonates that have disparate structures and polarities, which we hypothesize to affect the bulk modulus of the solution, and thus the acoustic response.^29^ Sensitivity of *K*_s_ to cosolvent composition would confirm the feasibility of operando acoustic additive-consumption tracking—for example, of fluoroethylene carbonate (FEC)—which remains an open question in battery-aging research.^30^

Here we report ultrasonic pulse–echo time-of-flight and densitometric measurements for ternary mixtures of LiPF_6_ in propylene carbonate (PC) and ethyl methyl carbonate (EMC) across a range of temperatures. Rather than the more widely used ethylene carbonate, we choose PC as a model cyclic carbonate because it is fully miscible in EMC, allowing us to access the entire spectrum of cosolvent ratio. Measured densities and speeds of sound are reported alongside calculated bulk moduli. A correlation that yields bulk modulus as a function of salt composition, solvent ratio, and temperature within the range of compositions studied is also developed using the symbolic regression technique.

Knowledge about compressibility furthers the understanding of bulk-solution microstructure.^31^ The data we report can also be used in conjunction with acoustic measurements of electrode composites or full cells to refine the electro-chemo-mechanical models used to study lithium-ion batteries.

## Experimental Section

### Electrolyte Preparation

Table 1 lists the supplier specifications of the LiPF_6_, PC, and EMC precursor materials used for this study. Solutions were prepared gravimetrically using an analytical balance with uncertainty of 0.2 mg (EX124; OHAUS) inside an argon-filled glovebox (Inert Technologies) with subppm levels of H_2_O and O_2_. The PC and EMC solvents were stored over 3 Å molecular sieves to keep the moisture content below 10 ppm, as confirmed by Karl Fischer titration.

**Table 1:**
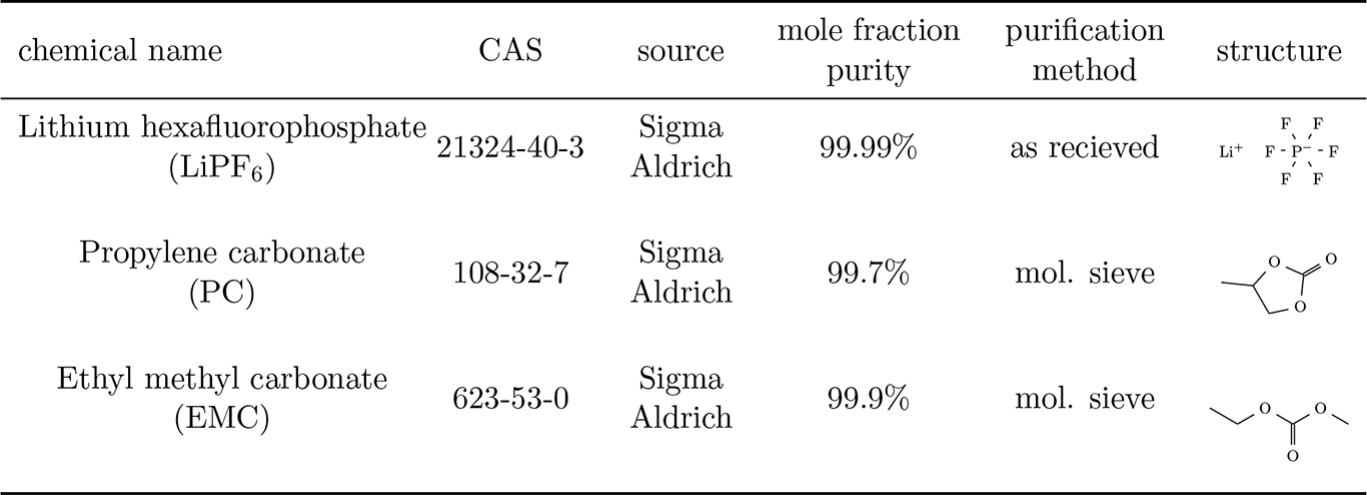
Details about Precursor Chemicals Used to Make Solutions

A comparison of the measured density and sound speed with available literature values for neat PC and EMC solvents is summarized in Table 2. Reported values of the relative permittivities of both solvents are also included.

**Table 2:** Physical Properties of Solvents at *T* = 298.15 K: Density (ρ), Speed of Sound (*c*), and Relative Permittivity (ε_r_)

	ρ/kg·m^–3^		*c*/m·s^–1^		ε_r_
Chemical	exp.	lit.	exp.^a^	lit.	lit.
PC	1200.0	1199.8^32^	1442	1442.3^34^	64.92^29^
		1199.7^33^		1443.4^35^	
EMC	1007.1	1007.0^32^	1169	N/A	2.958^29^
		1007.1^36^			

a Values interpolated from the reported experimental data.

**Figure 1:**
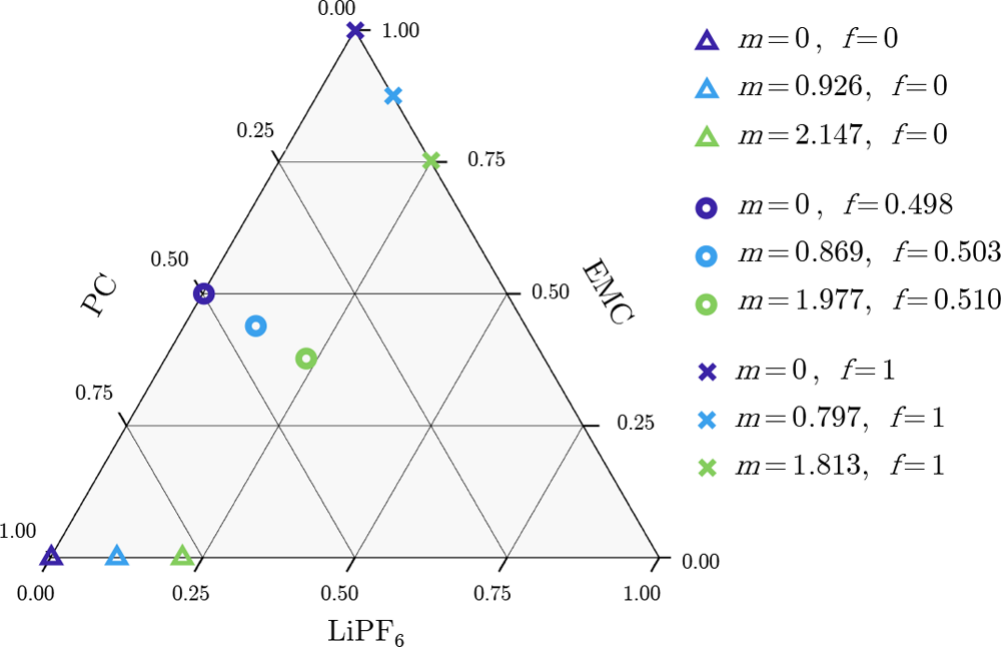
Ternary diagram depicting the mass fractions of LiPF_6_, PC, and EMC for the nine solutions tested in this study. The corresponding salt molality *m* in mol·kg^–1^ and fraction of PC in the neat cosolvent *f* are also stated in the legend.

In total, nine electrolyte compositions were tested, spanning three LiPF_6_ salt molalities from 0 to ∼2 mol.kg^–1^ and three solvent ratios: pure PC, pure EMC, and a cosolvent with ∼1:1 PC:EMC mass ratio. These compositions are depicted on the ternary chart in Figure 1. Points on the chart illustrate test-solution LiPF_6_, PC, and EMC mass fractions, denoted ω_LiPF_6__, ω_PC_, and ω_EMC_, respectively. For each composition in the ternary space, the legend also lists the corresponding salt molality *m*, given by
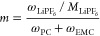
2in which *M*_LiPF_6__ = 151.905 g·mol^–1^ represents the molar mass of the salt, as well as the fraction of PC in the neat PC:EMC cosolvent *f*, defined as
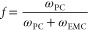
3Note that the mass fractions are constrained such that ω_LiPF_6__ + ω_PC_ + ω_EMC_ = 1, so the denominators in eqs 2 and 3 are less than unity when salt is present.

### Densitometry

The density ρ of each LiPF_6_:PC:EMC solution was measured using a high-precision oscillating U-tube density meter (DMA4100, Anton Paar) at ambient atmospheric pressure and temperatures *T* of 283.15, 293.15, 298.15, 303.15, 313.15 K. An inbuilt Peltier thermostat with 0.005 K precision maintained temperature control. The density meter was calibrated using ultrapure water (Type 1, Direct-Q 5UV, Millipore) and ethanol (≥99.5%, Sigma-Aldrich), and measurement uncertainty was found with *u*(ρ) = 0.2 kg m^–3^. Further details about the densitometric measurement technique were provided by Hou and Monroe.^33^

### Acoustic Measurements

**Figure 2:**
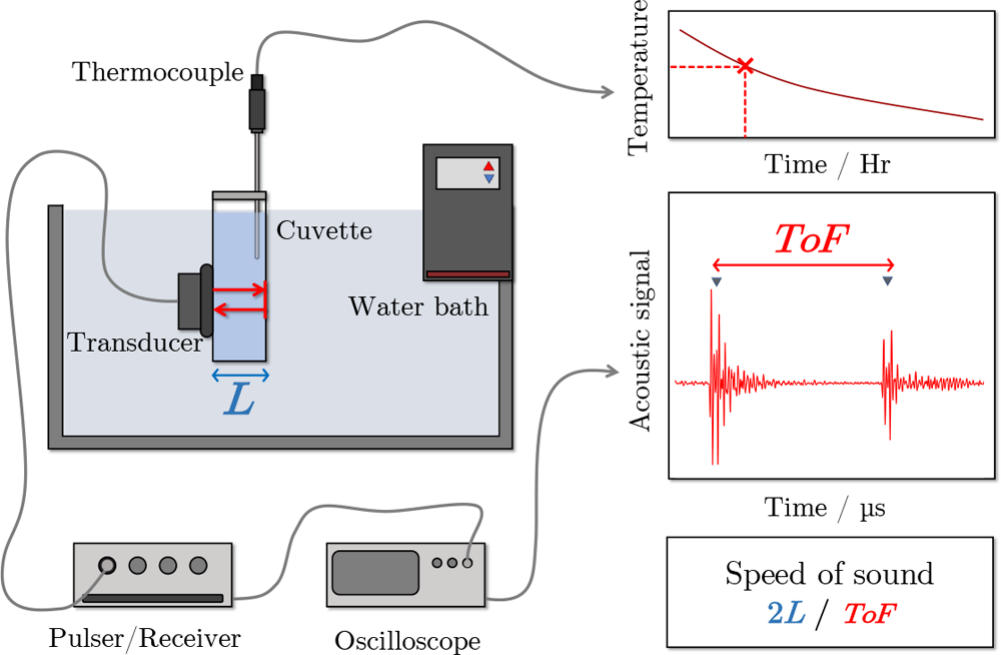
Schematic diagram of the temperature-controlled speed-of-sound experimental setup with labeled components. During the extended 4 h heating and cooling ramps, time-of-flight ToF for the acoustic pulse and echo is taken across the path length *L* of the sealed quartz cuvette containing the electrolyte sample. The sound speed *c* is then calculated as *c* = 2*L*/ToF. A typical collected waveform is depicted in full in Figure S1 of the Supporting Information.

A schematic diagram of the apparatus for acoustic measurements is provided in Figure 2. Approximately 10 mL of each sample solution was pipetted into a quartz cuvette (Macrocell 100-QS, Hellma) with a nominal path length of 9.5 mm. The sound speed in the sample at ambient pressure was determined by measuring the time-of-flight of acoustic waves across the cell with a single 5-MHz contact transducer (V109, Olympus NDT, Waltham, MA, USA) in pulse–echo mode. The transducer was coupled to the cell using acoustic coupling gel and an adjustable clamp (PM4; ThorLabs). A pulser-receiver (DPR300; Imaginant Inc., Pittsford, NY, USA) was used to generate the negative spike impulse excitation signal (10–70 ns, 100 V typ.) to the transducer, resulting in an acoustic signal approximately 0.3 μs in duration. The echo signal was amplified (−13 to 66 dB) by the pulser-receiver and the received waveform containing multiple reverberations was digitized at a sample rate of 100 MS s^–1^ using a USB-connected oscilloscope (HS5; TiePie Engineering, Sneek, The Netherlands). The difference in acoustic impedance between the sample liquid solution and cuvette walls resulted in a strong reflection, ensuring consistent sound-speed measurements.

To extract consistent times of flight from waveforms like those shown on Figure 2, each echo signal was cross-correlated using the Signal Processing Toolbox in MATLAB. The duration between the first and second echo signals calculated from the number of lags separating the autocorrelation peaks, as plotted in Supporting Information (SI) Figure S2, was then paired with the total distance traveled by the pulse and echo to extract the longitudinal speed of sound in the liquid sample.

Temperature control was maintained by placing the samples in a water-bath circulator (A45 HC, Thermo Scientific) with stability of 0.01 K. Each cuvette was sealed with a Teflon lid and high-vacuum grease (Corning) before being immersed up to the internal fluid level and clamped into place. The temperature of the sample was monitored with a stainless-steel sheathed K-type thermocouple probe (RS Pro) with accuracy *u*(*T*) = 1 K, immersed in the solution sample and secured in place via a port through the cuvette lid. External convection of heat by the water-bath circulation ensured a uniform temperature profile throughout the cuvette. Temperature readings (NI USB-9162) were recorded alongside oscilloscope data.

For each sample, measurements were taken periodically as the water bath temperature was ramped between 278.15 and 318.15 K at a rate of 10 K hr^–1^. An example of the thermal response is plotted in Figure S3 of the SI. This approach was first used to calibrate the path length *L* to 9.42 mm and *u*(*L*) = 0.02 mm with measurements using ultrapure water (Type 1, Direct-Q 5UV, Millipore). Recorded sound speeds for ultrapure water are provided in Figure S4. The use of slowly sweeping sample temperatures during the experiments did not induce appreciable hysteresis: sound speeds measured during heating and cooling (shown in Figure S5) differed by less than 2 m s^–1^. Similarly, analysis of runs repeated in triplicate (Figure S6) indicated a standard deviation of 2.5 m s^–1^. Combined uncertainties in sound speed measurements correspond to a relative percentage of 0.2%. Full waveforms recorded during the pulse–echo experiments, as well as corresponding sound-speed and temperature measurements, are included in the accompanying data repository.^37^

## Results and Discussion

### Density and Sound Speed

**Figure 3:**
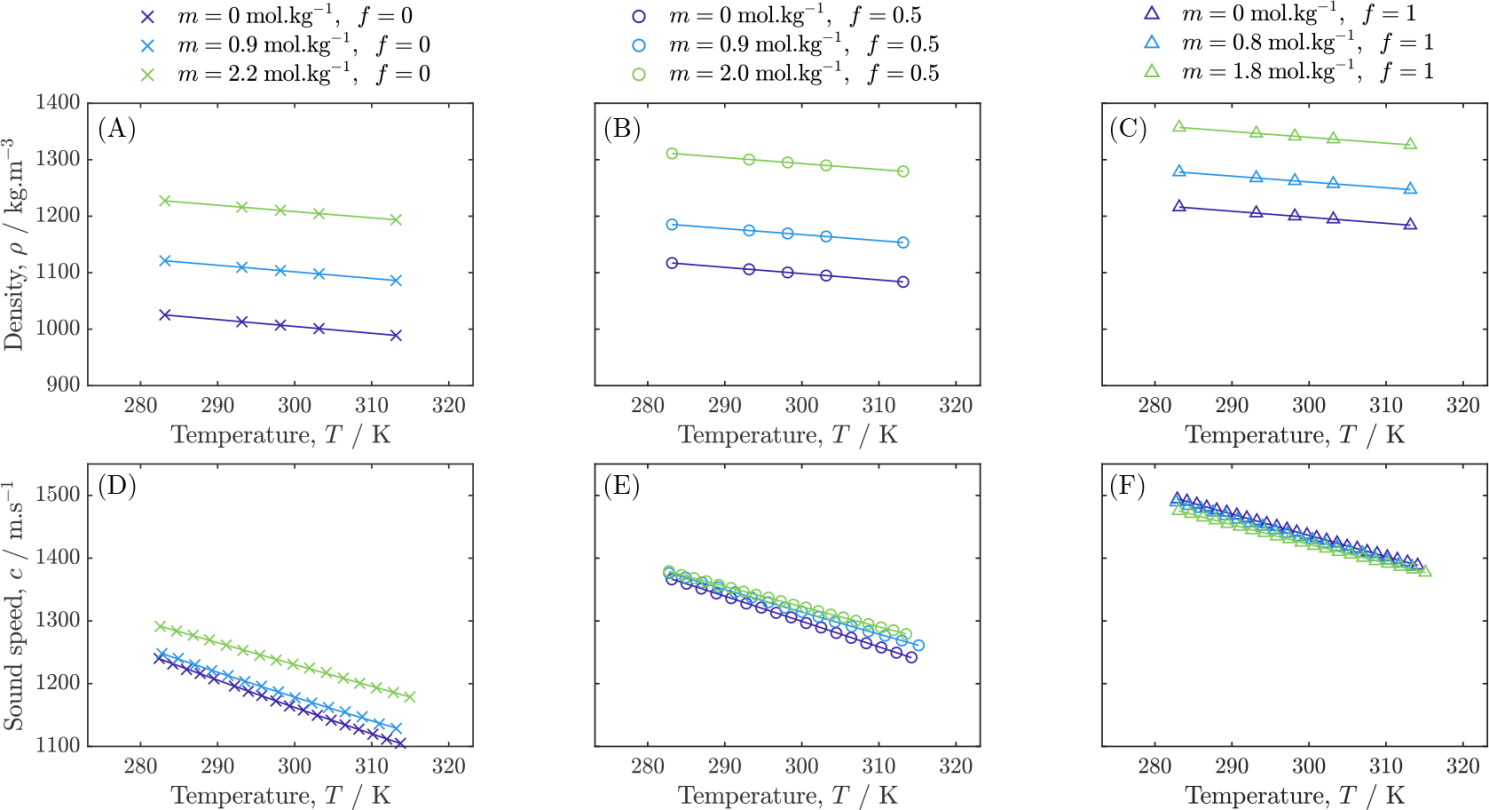
Densities ρ (A, B, C) and speeds of sound *c* (D, E, F) for LiPF_6_:PC:EMC ternary electrolyte solutions. Uncertainties fall within the marker size.

The measurements for solution density ρ and speed of sound *c* versus temperature *T* are presented in Tables 3, 4, 5, and 6, and plotted in Figure 3. Both ρ and *c* decrease as *T* increases. Within the salt and solvent composition range tested, density of the solutions varies in an approximately linear fashion, increasing as both the LiPF_6_ salt and denser PC solvent are added to the EMC solvent. Similar trends were observed with respect to *T*, *m*, and *f* in previous densitometric studies of LiPF_6_ in PC and EMC, as well as EC:EMC ternary systems.^33,36^ Separate measurements reported in the literature for LiPF_6_:EMC densities at a concentration of 1 mol·L^–1^ also align within 1% (Figure S7).^38^

**Table 3:** Experimental Values for the Densities of LiPF_6_ Salt Solutions with Molality *m* in PC:EMC Solvent Mixtures with Neat PC Mass Fractions *f* at Temperatures *T* = 283.15–313.15 K and Standard Atmospheric Pressure^a^

*m*/mol kg^–1^ = 0		*m*/mol kg^–1^ = 0.926		*m*/mol kg^–1^ = 2.147	
*f* = 0		*f* = 0		*f* = 0	
*T*/K	ρ/kg m^–3^	*T*/K	ρ/kg m^–3^	*T*/K	ρ/kg m^–3^
283.15	1025.2	283.15	1121.1	283.15	1227.2
293.15	1013.1	293.15	1109.5	293.15	1216.0
298.15	1007.1	298.15	1103.7	298.15	1210.5
303.15	1001.0	303.15	1097.9	303.15	1204.4
313.15	988.9	313.15	1086.1	313.15	1193.7

*m*/mol kg^–1^ = 0		*m*/mol kg^–1^ = 0.869		*m*/mol kg^–1^ = 1.977	
*f* = 0.498		*f* = 0.503		*f* = 0.510	
*T*/K	ρ/kg m^–3^	*T*/K	ρ/kg m^–3^	*T*/K	ρ/kg m^–3^
283.15	1117.1	283.15	1185.3	283.15	1311.1
293.15	1106.0	293.15	1174.7	293.15	1300.4
298.15	1100.4	298.15	1169.4	298.15	1295.2
303.15	1094.9	303.15	1164.1	303.15	1289.9
313.15	1083.7	313.15	1153.4	313.15	1279.4

*m*/mol kg^–1^ = 0		*m*/mol kg^–1^ = 0.797		*m*/mol kg^–1^ = 1.813	
*f* = 1		*f* = 1		*f* = 1	
*T*/K	ρ/kg m^–3^	*T*/K	ρ/kg m^–3^	*T*/K	ρ/kg m^–3^
283.15	1216.1	283.15	1278.1	283.15	1357.2
293.15	1205.4	293.15	1267.7	293.15	1346.7
298.15	1200.0	298.15	1262.5	298.15	1341.5
303.15	1194.7	303.15	1257.3	303.15	1336.4
313.15	1184.0	313.15	1247.1	313.15	1326.2

a Standard uncertainties *u* are *u*[*T*] = 5 mK, *u*[*m*] = 0.14 mmol/kg, and *u*[*f*] = 1.2 × 10^–5^. Expanded uncertainty (*k* = 2) *U*_c_[ρ] = 0.2 kg m^–3^.

**Table 4:** Experimental Sound Speeds *c* for LiPF_6_:EMC Solutions with Various Molalities *m* and Absolute Temperatures *T*, at Standard Atmospheric Pressure^a^

*m*/mol kg^–1^ = 0		*m*/mol kg^–1^ = 0.926		*m*/mol kg^–1^ = 2.147	
*f* = 0		*f* = 0		*f* = 0	
*T*/K	*c*/m s^–1^	*T*/K	*c*/m s^–1^	*T*/K	*c*/m s^–1^
282.41	1240	282.77	1248	282.55	1291
284.18	1231	284.89	1240	284.67	1284
285.96	1223	287.04	1230	286.82	1277
287.74	1216	289.22	1221	288.96	1270
289.51	1208	291.36	1213	291.12	1261
292.18	1196	293.52	1203	293.28	1253
293.98	1188	295.69	1196	295.47	1245
295.75	1181	297.88	1187	297.60	1237
297.56	1172	300.05	1177	299.77	1231
299.34	1164	302.22	1169	301.92	1225
301.13	1158	304.39	1162	304.11	1218
302.94	1149	306.56	1155	306.31	1209
304.73	1142	308.76	1147	308.45	1201
306.55	1134	310.96	1136	310.60	1193
308.33	1127	313.18	1129	312.81	1186
310.14	1119			314.95	1179
311.93	1112				
313.73	1105				

a Standard uncertainties *u* are *u*[*T*] = 1 K, *u*[*m*] = 0.14 mmol/kg, and *u*[*f*] = 1.2 × 10^–5^. Expanded uncertainty (*k* = 2) *U*_c_[*c*] = 3 m s^–1^.

**Table 5:** Experimental Sound Speeds *c* for Solutions of LiPF_6_ in PC:EMC Solvent Mixtures of Approximately 1:1 Mass Ratio, *f* ≈ 0.5, with Various Molalities *m* and Absolute Temperatures *T*, at Standard Atmospheric Pressure^a^

*m*/mol kg^–1^ = 0		*m*/mol kg^–1^ = 0.869		*m*/mol kg^–1^ = 1.977	
*f* = 0.498		*f* = 0.503		*f* = 0.510	
*T*/K	*c*/m s^–1^	*T*/K	*c*/m s^–1^	*T*/K	*c*/m s^–1^
282.16	1370	282.76	1376	282.76	1379
284.07	1363	284.90	1369	284.37	1373
286.00	1355	287.04	1361	285.99	1368
287.93	1348	289.18	1353	287.60	1363
289.86	1340	291.34	1346	289.21	1357
291.80	1331	293.48	1337	290.81	1352
293.77	1325	295.63	1330	292.44	1347
295.70	1316	297.80	1321	294.05	1342
297.64	1308	299.99	1315	295.67	1337
299.58	1300	302.16	1307	297.28	1331
301.54	1293	304.30	1298	298.90	1326
303.50	1286	306.46	1291	300.52	1321
305.45	1276	308.63	1283	302.14	1316
307.37	1269	310.80	1276	303.76	1310
309.33	1261	312.98	1269	305.38	1306
311.31	1253	315.16	1261	307.02	1300
313.27	1245			308.64	1295
				310.28	1290
				311.89	1285
				313.53	1279

a Standard uncertainties *u* are *u*[*T*] = 1 K, *u*[*m*] = 0.14 mmol/kg, and *u*[*f*] = 1.2 × 10^–5^. Expanded uncertainty (*k* = 2) *U*_c_[*c*] = 3 m s^–1^.

**Table 6:** Experimental Sound Speeds for LiPF_6_:PC Solutions with Various Molalities *m* and Absolute Temperatures *T*, at Standard Atmospheric Pressure^a^

*m*/mol kg^–1^ = 0		*m*/mol kg^–1^ = 0.797		*m*/mol kg^–1^ = 1.813	
*f* = 1		*f* = 1		*f* = 1	
*T*/K	*c*/m s^–1^	*T*/K	*c*/m s^–1^	*T*/K	*c*/m s^–1^
282.90	1494	282.76	1489	283.08	1475
284.16	1491	284.36	1483	284.66	1471
285.44	1486	285.95	1479	286.24	1465
286.73	1481	287.56	1473	287.85	1460
288.02	1476	289.16	1467	289.41	1455
289.33	1473	290.75	1462	291.02	1450
290.63	1468	292.39	1456	292.59	1445
291.93	1464	293.99	1450	294.21	1440
293.23	1459	295.59	1445	295.80	1435
294.51	1455	297.21	1439	297.42	1431
295.82	1450	298.82	1434	299.01	1425
297.14	1446	300.42	1429	300.63	1420
298.41	1441	302.05	1423	302.22	1415
299.73	1437	303.66	1418	303.82	1410
300.99	1433	305.28	1413	305.41	1406
302.28	1428	306.91	1408	307.01	1401
303.64	1424	308.50	1402	308.62	1396
304.95	1419	310.11	1397	310.25	1391
306.26	1415	311.75	1391	311.86	1386
307.55	1410	313.37	1386	313.48	1382
308.84	1407			315.10	1377
310.16	1402				
311.47	1398				
312.78	1393				
314.10	1388				

a Standard uncertainties *u* are *u*[*T*] = 1 K, *u*[*m*] = 0.14 mmol/kg, and *u*[*f*] = 1.2 × 10^–5^. Expanded uncertainty (*k* = 2) *U*_c_[*c*] = 3 m s^–1^.

Sound speed within the electrolytes tested depends strongly on the pure-solvent properties. The speed of sound in PC is faster than that in EMC, which is expected both because of PC’s higher dipole moment (Table 2) and its more rigid, lower-branching molecular geometry. Higher polarity and lower branching solvents produce solutions with molecules in closer contact, providing a better medium for propagating sound.^39^ The binary LiPF_6_:PC solutions exhibit very little variation in speed of sound with respect to salt content, whereas in solutions containing EMC, sound speed increases with salt content as higher-order solution structures form. This observation aligns with PC and EMC’s contrasting dielectric properties outlined in Table 2: in previous Walden analyses, it was shown that the relationship between ionic conductance and viscosity is upheld in LiPF_6_:PC, qualitatively indicating a high ionicity.^36^ On the other hand, LiPF_6_:EMC demonstrated characteristics of a relatively weak electrolyte, especially at lower salt concentrations.

### Bulk Modulus

**Figure 4:**
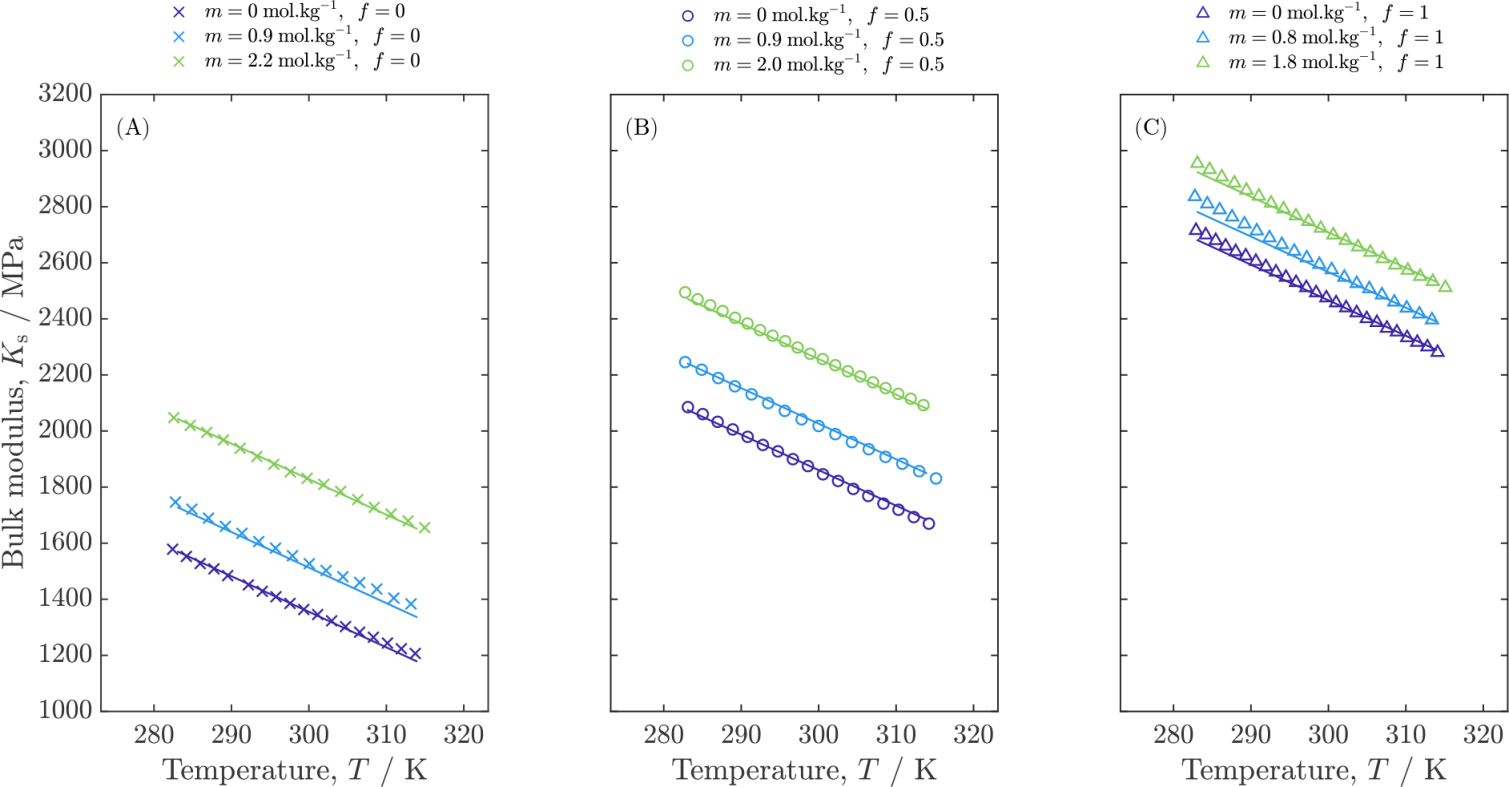
Bulk modulus *K*_s_ as a function of temperature and composition for LiPF_6_ electrolytic solutions in EMC (A), PC:EMC (B), and PC (C). Curves represent the correlation presented in eq 4.

Structural understanding of electrolytic solutions can be developed by examining the composition dependence of their bulk moduli. The isentropic bulk modulus *K*_s_ of the samples was determined by using the above results for density and speed of sound in eq 1, producing a relative uncertainty associated with the calculation of 0.3%. Calculated isentropic bulk moduli for the LiPF_6_:PC:EMC solutions tested are plotted as a function of temperature in Figure 4. In line with the approximately linear variation of density with temperature in the range tested, the densities of electrolytic solutions with given *m* and *f* corresponding to the temperatures of their sound-speed measurements were determined by linear interpolation between the data points in Table 3.

Bulk modulus *K*_s_ generally provides a measure of the pressure required to produce a fractional change in volume for a solution at a given temperature and composition. All the *K*_s_ values presented here decrease monotonically with increasing temperature. Clear trends are also observed with respect to salt molality and solvent fraction. PC’s rigid ring structure provides fluid samples with more resistance to uniform compression than EMC’s linear-carbonate geometry.^40^ Similarly, PC’s higher polarity and resulting higher strength of dipole-dipole interactions suggest a more compact solvent configuration, making *K*_s_ higher. The moduli of the 1:1 PC:EMC samples appear to follow a relatively simple mixing rule, lying about midway between the neat-solvent properties at all temperatures. Across all three solvent ratios, the addition of LiPF_6_ led to a concomitant rise in *K*_s_, with an increasing sensitivity to solute molality observed as the neat-solvent fraction of EMC rose. Rigidity-promoting secondary and tertiary solvation structures are expected to become more prevalent as salt content increases. Ion–solvent coordination in concentrated lithium-ion electrolytes is a well-documented phenomenon, with bound solvent making up a significant portion of the overall solvation structure.^41^ During typical lithium-ion battery operation, an equilibrium electrolyte composition of 1 mol kg^–1^ can be polarized to reach 0 and 2 mol kg^–1^ across the solution domain. In the case of 1:1 PC:EMC, this represents a change in bulk modulus *K*_s_ of approximately 30% at 298.15 K. Similar variability in *K*_s_ is also observed across the practical temperature range probed in this work.

The data presented in Figure 4 show that *K*_s_ can be seen as a suitable proxy for multicomponent electrolyte composition. Acoustic measurements could augment standard methods of composition assay, which tend to be based on more elaborate spectroscopic techniques. Acoustic data could be particularly useful in situations where measurements of density alone do not suffice to specify composition.^42^ Trends in bulk moduli can also help to elucidate microscopic solvation states and thermodynamic data for lithium-ion electrolytes, as has been done for common aqueous systems.^31,43^

### Correlation for Bulk Modulus

To facilitate the use of data from this paper in computational studies, a correlation for the isentropic bulk modulus *K*_s_, based on the data reported above, was developed using the symbolic regression technique and the dimensionless variables of molality, temperature, and PC solvent fraction. The correlation chosen based on accuracy and parsimony was found to be
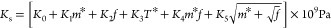
4in which input variable m* is the unitless salt molality (m* = *m*/(1 mol kg^–1^)) and T* is the unitless absolute temperature (T* = *T*/(1 K)). The fitted parameters *K*_*i*_ are provided in Table 7. Note that the output bulk modulus *K*_s_ in eq 4 is scaled to have units of Pa, while its input variables (m*, T*, *f*) and coefficients in Table 7 are unitless. The correlation has a goodness-of-fit (*R*^2^) of 0.999, and a root-mean-squared error (RMSE) across the *K*_s_ of 0.015 GPa, corresponding to a root-mean-squared percentage error of ∼1%.

**Table 7:** Unitless Parameters for the Bulk Modulus *K*_s_ Correlation in eq 4

Parameter	Value
*K*_0_	5.2990
*K*_1_	0.3160
*K*_2_	1.2512
*K*_3_	– 0.0131
*K*_4_	– 0.1300
*K*_5_	– 0.1402

The method chosen to develop this expression—symbolic regression (SR)—is a supervised machine learning technique that can be used to co-optimize specific functional forms and parameter values to fit the available data set.^44,45^ Unlike black-box regression techniques, expressions given by SR are readily explained. SR also has the benefit of generating accurate functions that tend to be simpler than the fits yielded by purely statistical approaches such as higher-order multivariate polynomial methods.

We followed the SR approach implemented by Flores et al., originally used to develop correlations for lithium-ion electrolyte conductivity.^46^ The full *K*_s_ set of 168 data points was split into ten testing and training groups for cross validation. The SR algorithm was then run on the training sets, searching through combinations of √, ^∧^1, ^∧^2, and ^∧^3 operators. The selected formula was chosen based on several criteria: maximizing *R*^2^ and minimizing RMSE across both training and test sets, while maintaining parsimony in the number of terms in the expression and consistency with regard to the number of occurrences of the same set of terms that were discovered by the algorithm across all training sessions. Once the set of terms was chosen, the final parameters were generated by fitting all available *K*_s_ data.

A full description of the SR approach is detailed in the SI, discussion section S4. A comparison with linear and polynomial fitting techniques is included in Table S3. Results from the cross-validation and expression search with feature engineering steps and expression sets are also reported in Figures S8 and S9 and Tables S1 and S2.

## Conclusion

The isentropic bulk modulus *K*_s_ of solutions of LiPF_6_ in mixtures of cyclic (PC) and linear (EMC) carbonate solvents has been measured as a function of ternary composition and temperature. The electrolytic solutions studied here were intended to be representative of commercially ubiquitous ternary formulations of lithium electrolytes. A correlation that fits the data well with respect to salt molality, solvent ratio, and temperature within the ranges probed was developed. Bulk moduli were calculated by combining temperature-controlled experimental densitometry data with sound speeds measured by acoustic time-of-flight. The bulk modulus was found to vary from 1–3 GPa across the composition and temperature ranges tested here, which are pertinent to conventional lithium-ion batteries. Trends in the composition and temperature dependence of an electrolyte’s isentropic modulus (i.e., its compressibility) can be rationalized by considering bulk solvent structures and the degree of solute–solvent coordination. Thus, acoustical studies provide a straightforward and cost-effective route by which to understand thermodynamic states and microscopic solvation environments in novel electrolyte formulations. Isentropic bulk modulus may also serve as parameter that can be used to rapidly diagnose additive consumption, to aid battery lifetime prediction. This acoustic data for cosolvated lithium-ion electrolytes is intended to motivate investigations of more complex electrolyte systems involving additives. The baseline set of electrolyte-specific information reported here could be used in the future to elucidate the structures of porous-electrode domains with ultrasound.

## Supporting Information Available

Recorded acoustic wave forms and cross-correlation signal processing (Figures S1 and S2); temperature heating and cooling ramp (Figure S3); calibration curve for ultrapure water (Figure S4); sound speed measurements on heating and cooling repeats (Figures S5 and S6); density comparison for LiPF_6_:EMC (Figure S7); training data and results from symbolic regression cross validation (Figures S8, S9, and Tables S1, S2, and S3) (PDF)
