# Supplementary material for: Compressibility of Lithium Hexafluorophosphate Solutions in Two Carbonate Solvents
Source: J Chem Eng Data. 2023 Mar 14;68(4):805–12. doi: 10.1021/acs.jced.2c00711 (PMC10108564; doi:10.1021/acs.jced.2c00711)
Supplement: Supplementary file 1 — je2c00711_si_001.pdf [file je2c00711_si_001.pdf]

Supporting Information

Compressibility of Lithium Hexafluorophosphate Solutions in Two Carbonate Solvents

Andrew A. Wang,[†,‡,¶] Delia Persa,[†,¶] Sara Helin,[†] Kirk P. Smith,[†] Jason L. Raymond,[†] and Charles W. Monroe[*,†,‡]

†*Department of Engineering Science, University of Oxford, Parks Road, Oxford, OX1 3PJ, UK*

‡*The Faraday Institution, Becquerel Avenue, Harwell Campus, Didcot, OX11 0RA, UK*

¶*Contributed equally to this work*

E-mail: *charles.monroe@eng.ox.ac.uk

S1 Acoustic signal processing

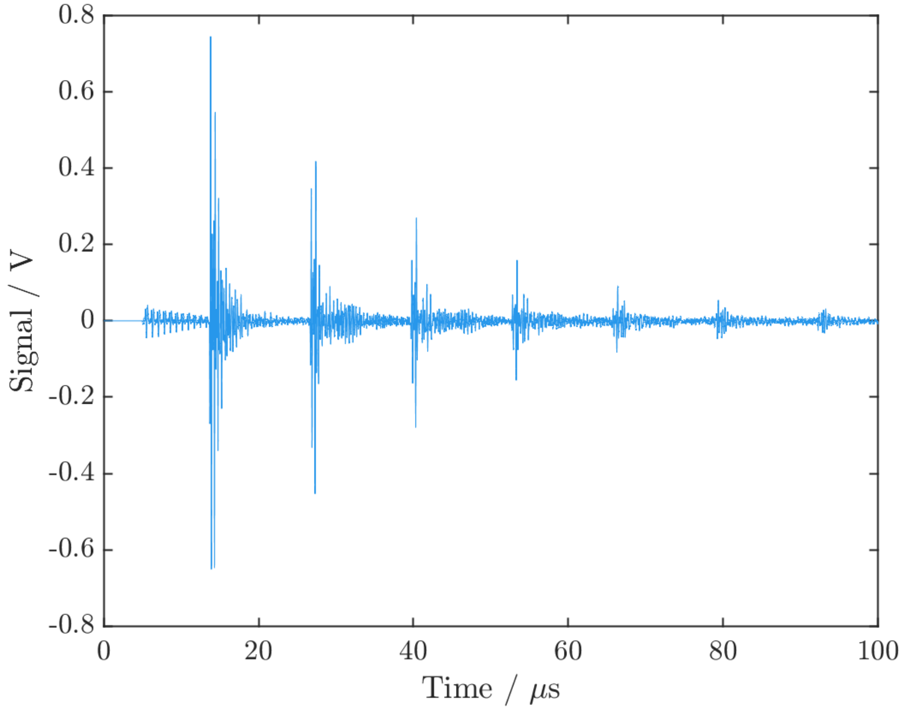

Figure S1: Example of an entire collected acoustic waveform, for 1 M $LiPF_6$:PC at room temperature. The first 5 μs of the main bang was removed from all waveforms when processing autocorrelations for calculating time-of-flight.

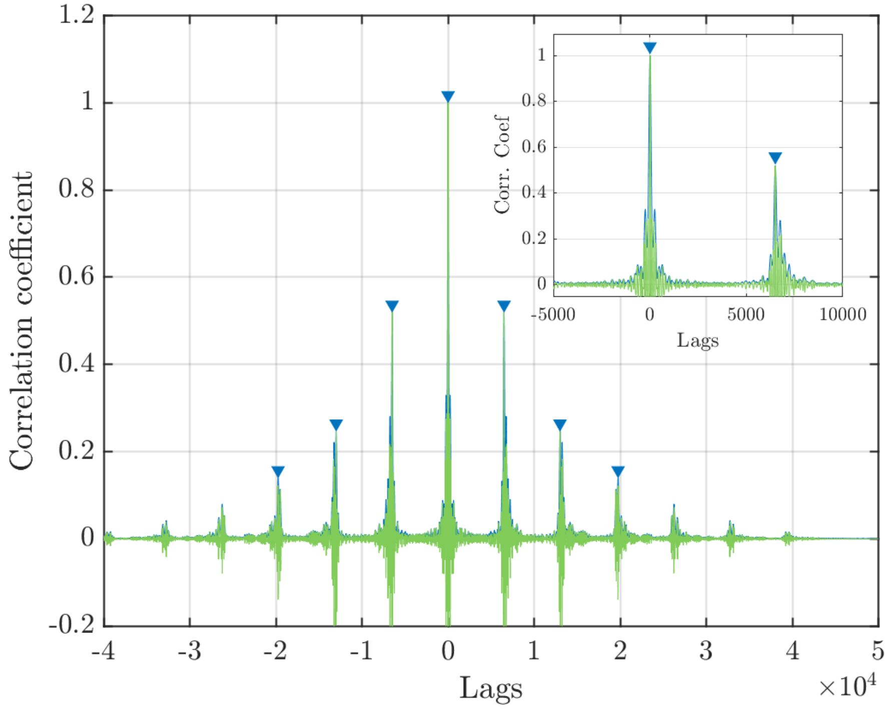

Figure S2: Example of cross-correlation signal processing for 1 M $LiPF_6$:PC at room temperature. The peaks indicate points where the highest degrees of similarity occur when the waveform vector is shifted with the time vector step size (lag). Enveloping of the wave forms allows for the primary and secondary peaks to be picked, such that a consistent time-of-flight can be measured across experimental samples.

S2 Speed of sound

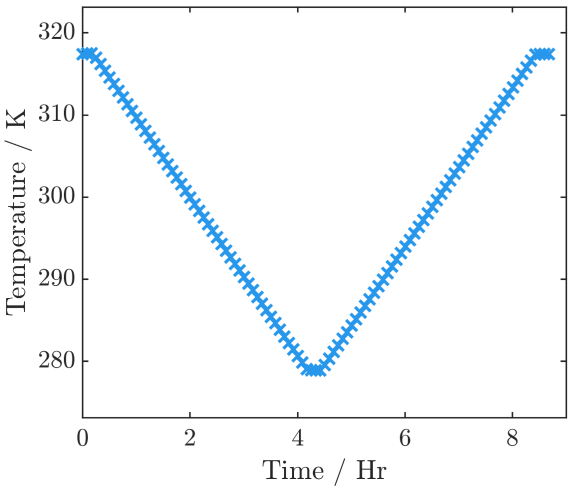

Figure S3: Thermocouple readings during the 4 hour cooling and heating ramp. The immersion circulator waterbath was programmed to sweep between 5 and 45 °C. Acoustic acoustic times-of-flight were measured for electrolyte samples at various bulk temperatures between 10 and 40 °C.

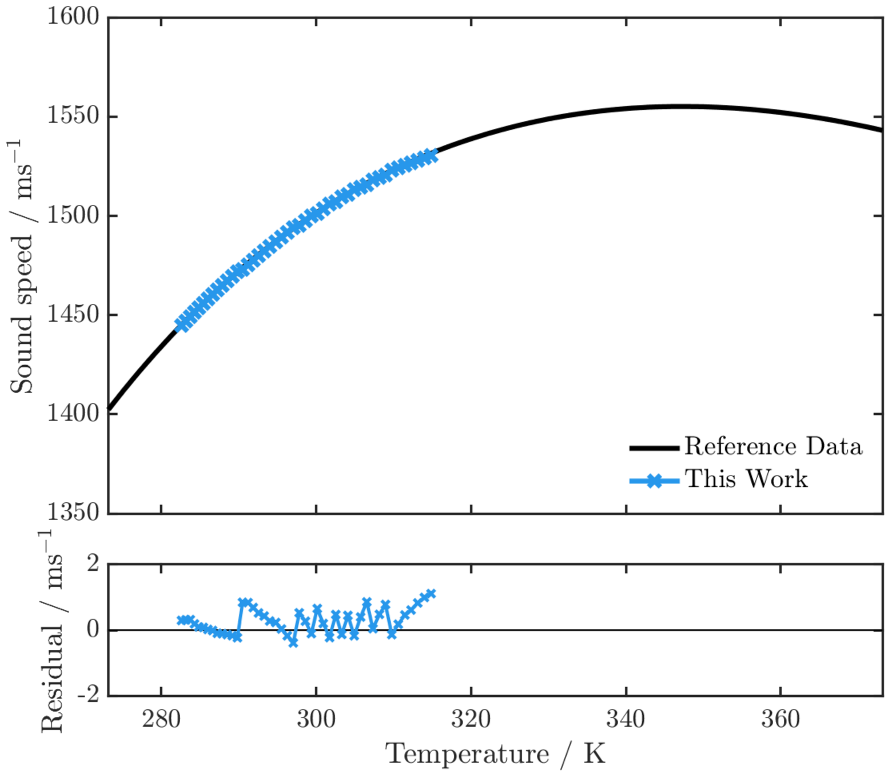

Figure S4: Comparison of sound-speed measurements for ultrapure water with literature data as a function of temperature, which was used to calibrate the path length of the quartz cuvette. Literature correlations are taken from Del Grosso et al. and Bilaniuk et al.[1,2] Residuals between the experimental and reference values are plotted in the lower panel.

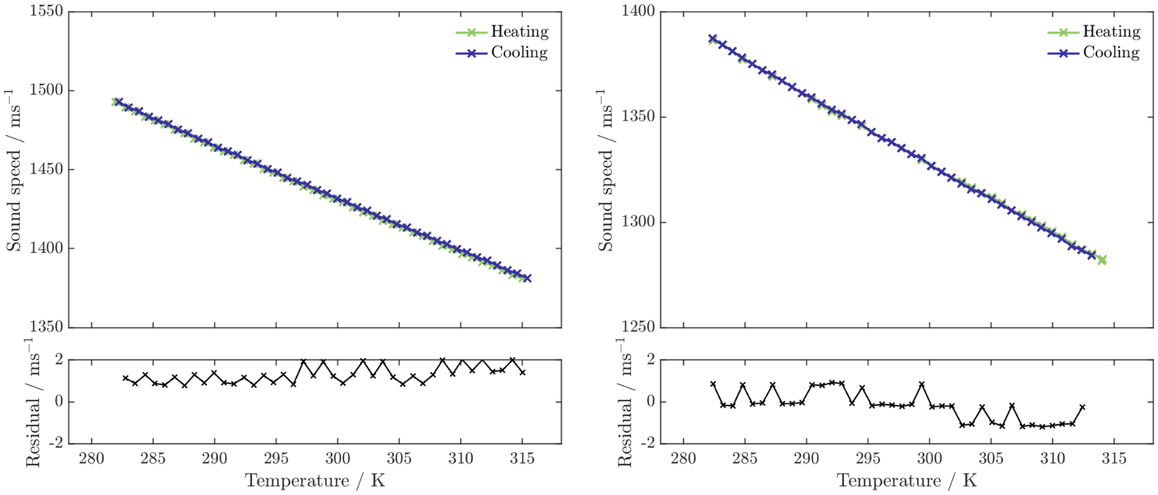

Figure S5: Speed of sound measured during heating (green) and cooling (blue) for ∼1 M $LiPF_6$ in PC (left) and ∼2 M $LiPF_6$ in 1:1 PC:EMC. Residuals show that the selected 4 hour thermal ramp suffices to provide a quasi-equilibrated thermal state for sound-speed measurement.

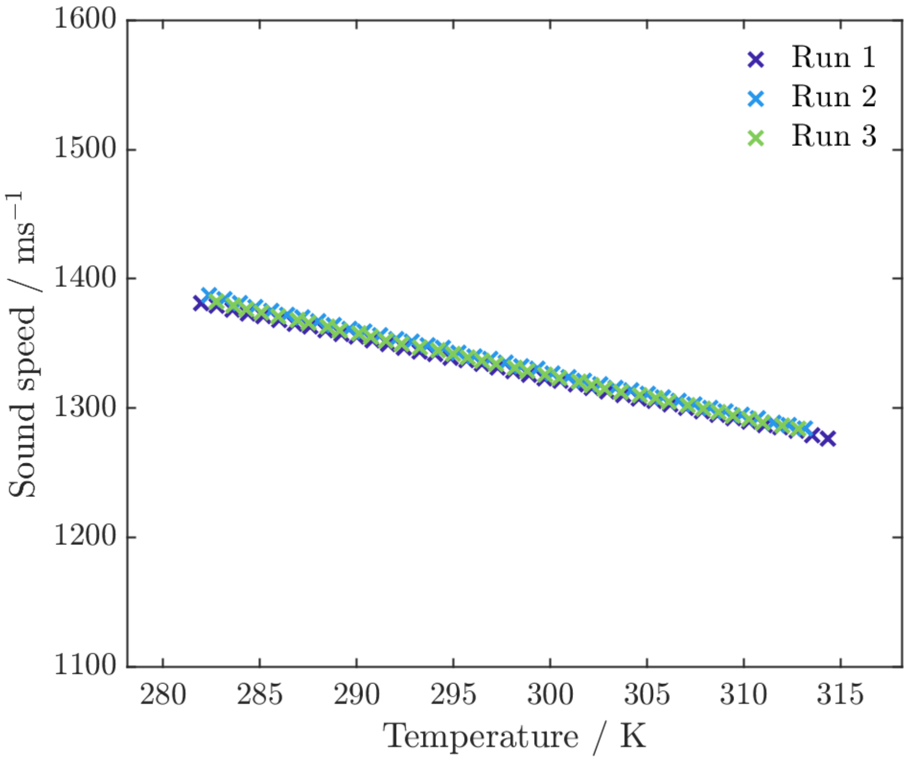

Figure S6: Triplicate of speed of sound measurements indicating repeatability of the acoustic experiment.

S3 Density comparison

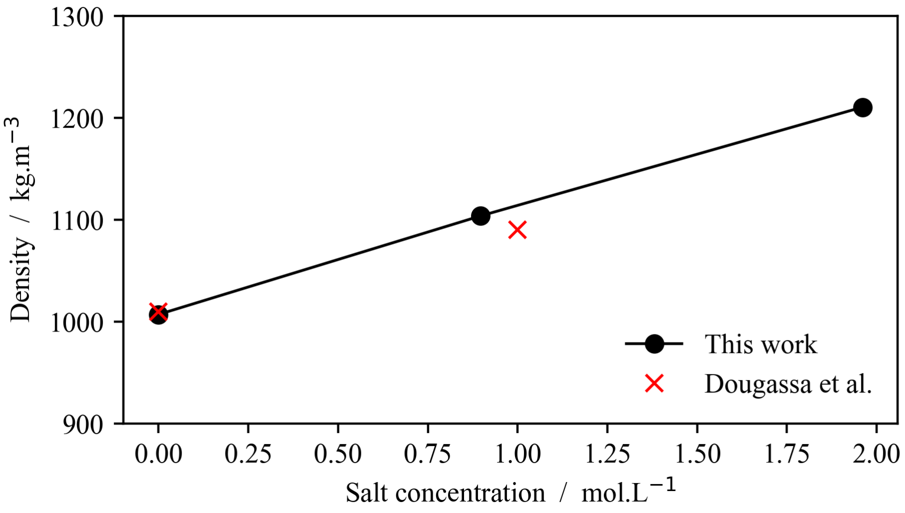

Figure S7: Density comparison for $LiPF_6$:EMC at 298.15 K reported in this work (black circles) and in Dougassa et al (red crosses).[3]

S4 Symbolic regression

Symbolic regression was implemented following the process laid out by Flores et al.[4] Here, the Autofeat library was used to generate and down-select features.[5] We use 3 feature engineering steps in this work, and a sparser set of operations ($\sqrt{}$, ^2, and ^3) because K_S was observed to vary relatively smoothly and monotonically. The abs(x) function was excluded to ensure that the correlation remained differentiable; sin(x), cos(x), and other trigonometric functions were also excluded on the basis that periodic behaviour is not expected. Further details of the SR process are available from the Flores paper,[4] as well as the online data repository that accompanies this article.[6]

Figures S8, S9 and Tables S1, S2 show how SR-discovered models were evaluated and downselected. Table S3 provides a comparison to benchmark polynomial and linear fitting correlations. The number of terms and goodness of fit for the 6-term SR correlation presented balances accuracy and simplicity within the uncertainty range of the measurements.

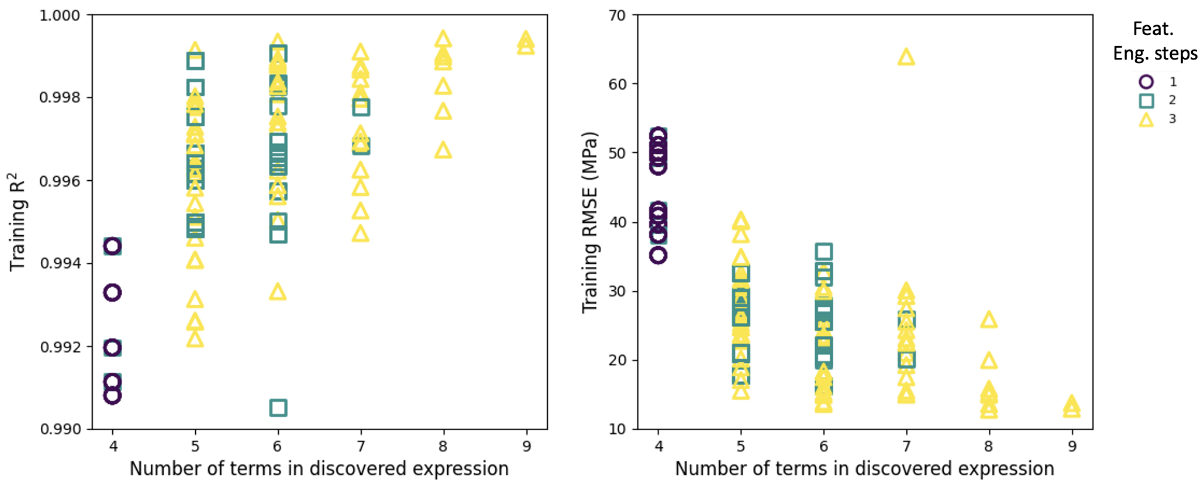

Figure S8: Accuracy vs parsimony of SR-discovered correlation expressions when compared to the cross-validation test sets. Each data point represents an expression whose shape and color indicates the number of feature engineering steps in its development.

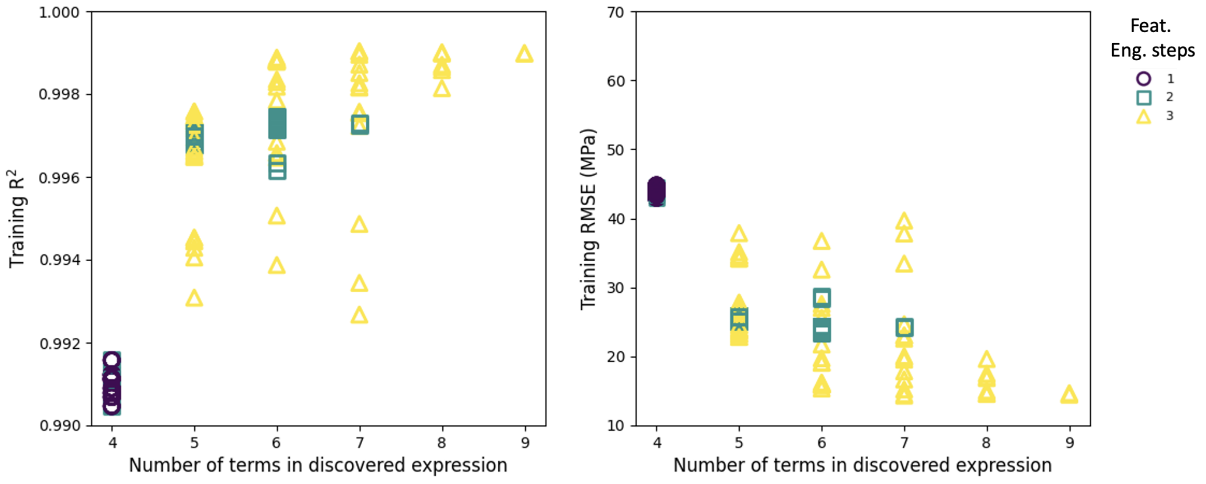

Figure S9: Accuracy vs parsimony of SR-discovered correlation expressions when compared to the cross-validation training sets. Each data point represents an expression whose shape and color indicates the number of feature engineering steps in its development.

Table S1: Ranking of symbolic regression cross-validation results based on the test R^2. While generally expressions with a higher number of terms achieve better fits, a particular expression set highlighted in gray shows a strong fit to the data while only requiring 6 terms. RMSE is given in units of GPa.

Expressions	Occur.	Terms	R^2_{test}	$RMSE_{test}$
(m - f**2)**2 , (sqrt(m) - f)**2 , T , m , m**2...	1	8	0.999418	0.012773
(-sqrt(m) + f**3)**2 , (sqrt(m) + f**3)**2 , T ...	1	9	0.999404	0.012921
(m - f**3)**2 , (sqrt(m) + f**3)**2 , T , m , m...	1	8	0.999057	0.013581
T , m , m*f , f , sqrt(m + sqrt(f))	9	6	0.999340	0.013599
(f**2 + f)**3 , (sqrt(m) + f**3)**2 , T , T**3*...	1	9	0.999239	0.013742
(m - f**3)**2 , (f**2 + f)**3 , T , m , m**15 ,...	1	8	0.998980	0.014943
(-m + f**3)**2 , (f**2 + f)**3 , T , m , m**6*f...	1	7	0.999099	0.014956
(m - f**2)**2 , T , m , m**2*sqrt(f) , r , sqrt...	1	7	0.998747	0.015043
(-m + f**3)**2 , (f**2 + f)**3 , (sqrt(m) + f**...	1	8	0.998275	0.015213
(m - f**3)**2 , (f**2 + f)**3 , T , m , m**9*f*...	1	7	0.998666	0.015525

Table S2: Ranking of 6-term symbolic regression cross-validation results based on their number of occurrences. The selected expression highlighted in gray shows the strongest fit to the data while also occurring a high proportion of times during the SR cross validation process. RMSE is given in units of GPa.

Expressions	Occur.	Terms	R^2_{test}	$RMSE_{test}$
T , m , m**3*f**3 , f , sqrt(m)*sqrt(f)	19	6	0.999060	0.016225
T , m , m*f , f , sqrt(m + sqrt(f))	9	6	0.999340	0.013599
(m**3 - f**3)**3 , T , m , f , sqrt(m + sqrt(f))	2	6	0.995038	0.029940
T , m , m**2*f**2 , f , sqrt(m)*sqrt(f)	2	6	0.998271	0.020722
T , m , m**2*f**2 , m**2*sqrt(f) , f	2	6	0.995004	0.032023
(-sqrt(m) + f**2)**2 , (sqrt(m) + f**2)**2 , T ...	1	6	0.995877	0.023522
(-sqrt(m) + f**2)**2 , T , m , f , sqrt(m)*f**2	1	6	0.997531	0.023241
T , T**3*m**9 , m , m**9*f**3 , f	1	6	0.998868	0.017811
(m - f**2)**2 , (sqrt(m) + f**2)**2 , T , f , s...	1	6	0.997283	0.024253
(-m + r)**2 , T , m , m**3*f , f	1	6	0.996227	0.025404

Table S3: Benchmark fitting correlations and their goodness of fit. RMSE is given in units of GPa.

Model	Formula	Terms	R^2	RMSE
Linear	$K_0 + K_1 m + K_2 f + K_3 T$	4	0.993	0.038
Polynomial	$K_0 + K_1 T + K_2 m + K_3 f + K_4 T m$ $+ K_5 m f + K_6 T r + K_7 T^2 + K_8 f^2 + K_9 m^2$	10	0.999	0.011

References

(1) Del Grosso, V. A.; Mader, C. W. Speed of Sound in Pure Water. *J. Acoust. Soc. Am.* **1972**, *52*, 1442–1446.

(2) Bilaniuk, N.; Wong, G. S. K. Speed of sound in pure water as a function of temperature. *J. Acoust. Soc. Am.* **1993**, *93*, 1609–1612.

(3) Dougassa, Y. R.; Tessier, C.; Ouatani, L. E.; Anouti, M.; Jacquemin, J. Low pressure carbon dioxide solubility in lithium-ion batteries based electrolytes as a function of temperature. Measurement and prediction. *J. Chem. Thermodyn.* **2013**, *61*, 32–44.

(4) Flores, E.; Wölke, C.; Yan, P.; Winter, M.; Vegge, T.; Cekic-Laskovic, I.; Bhowmik, A. Learning the laws of lithium-ion transport in electrolytes using symbolic regression. *Digital Discovery* **2022**, *1*, 440–447.

(5) Horn, F.; Pack, R.; Rieger, M. *Machine Learning and Knowledge Discovery in Databases*; Springer International Publishing, 2020; pp 111–120.

(6) Wang, A. A. Data repository, online at 'ndrewwang/SoundSpeed: repository'. *Zenodo* **2022**, https://doi.org/10.5281/zenodo.7081598.
